# Effect of Two Different Doses of Neostigmine on the Gastric Residual Volume and Aspiration in Critically Ill Patients Under Enteral Feeding; A Comparative Controlled Randomized Trial

**DOI:** 10.5812/aapm-158019

**Published:** 2025-02-17

**Authors:** Sameh Hamdy Abdelhamid Seyam, Ibrahim Elabd Hassan, Abdallah Elabd Hassan, Mostafa Mohamad Elsayed

**Affiliations:** 1 Anesthesiology, Intensive Care and Pain Management Department, Faculty of Medicine for Boys, Al-Azhar University, Cairo, Egypt; 2 Anesthesiology, Intensive Care and Pain Management Department, Faculty of Medicine, Aswan University, Aswan, Egypt

**Keywords:** Neostigmine, Gastric, Residual, Enteral, Aspiration, Feeding

## Abstract

**Background:**

Delayed gastric emptying increases the risk of patient morbidity in the ICU. Intensive care researchers have exerted considerable effort to measure and regulate gastric residual volumes (GRV) in ventilator-operated patients.

**Objectives:**

This study examines a cross-sectional, double-blind clinical trial designed to assess the effect of the addition of neostigmine to metoclopramide GRV in ICU patients and the risk of aspiration in those patients.

**Methods:**

Participants were categorized into three groups: Group I (n = 41) and group II (n = 43) received neostigmine 1 mg and 2 mg, respectively, and a control group (group III, n = 40) received 10 mL of normal saline. All participants received an intravenous administration of 10 mg of metoclopramide. The GRV was measured every 3 hours before enteral feeding. Aspiration through nasogastric (NG) or orogastric (OG) tubes was done before the next due bolus of feeding. The study did not receive any external funding support. The possessed data was interpreted using the PASS program, which set the alpha error at 5% and the power at 80%.

**Results:**

There was a significant variation among the three groups regarding the GRV. Metoclopramide used alone, did not profoundly alter the GRV at various time intervals. However, the administration of neostigmine resulted in a significant reduction in GRV at 3 and 6 hours post-injection. The GRV increased six hours post-injection, indicating that the drug combination resulted in a short-term effect. We did not observe any significant link between GRV and aspiration incidence, which happened even with low-volume aspirations. We used immunoassay to determine pepsin in the collected tracheal aspirations.

**Conclusions:**

Combining neostigmine and metoclopramide can effectively reduce GRV in ICU patients receiving enteral nutrition.

## 1. Background

Gastric motility disturbances are prevalent, with more than 50% of mechanically ventilated ICU patients exhibiting delayed gastric emptying (1). These disorders lead to high gross gastric residual volumes (GRV), which serve as an unfavorable indicator of potential jeopardy to the patient’s life (2). Delayed gastric emptying increases the risk of ventilator-associated pneumonia (VAP) and inadequate caloric intake, prolonging hospitalization and increasing patient risk. So, intensive care researchers have exerted considerable effort to measure and regulate GRV in ventilator-operated patients (3). Therefore, enhanced GRV management is crucial for promoting improved patient care (4). Various medications, including metoclopramide and neostigmine, have been utilized to enhance gastric emptying; nonetheless, comprehensive studies demonstrating the superiority of one agent over another are not extensively documented (5). 

There are two methods for addressing this issue: The surgical and pharmacologic approaches, each with its respective disadvantages (6). Metoclopramide, erythromycin, and cisapride have all been utilized in ICU patients. Nonetheless, none has demonstrated superiority over the others (7). Recent studies have examined neostigmine’s prokinetic properties. These clinical trials aim to determine the efficacy of neostigmine at various doses compared to metoclopramide on the GRV of ICU patients receiving enteral nutrition (8). 

Aspiration exactly is the most formidable and probably severe complication of enteral feeding. The detrimental effects of aspiration may have been overblown in previous studies (9). However, some aspirations may happen on a bigger than conventional prevalence; both mortality and morbidity are considerable risks and burdens resulting from it (10). However, aspiration of oral secretions may happen even more than gastric secretions. The extent to which aspiration of gastric secretions versus oral secretions leads to aspiration pneumonia is unclear. Scanty changes in the management of the enteral feeding procedure would be anticipated to affect the risk of aspiration of oro-pharyngeal contents (11). 

## 2. Objectives

This study examines a cross-sectional, double-blind clinical trial designed to assess the effect of the addition of neostigmine to metoclopramide on GRV in ICU patients and the risk of aspiration in those patients.

## 3. Methods

**Figure 1.**
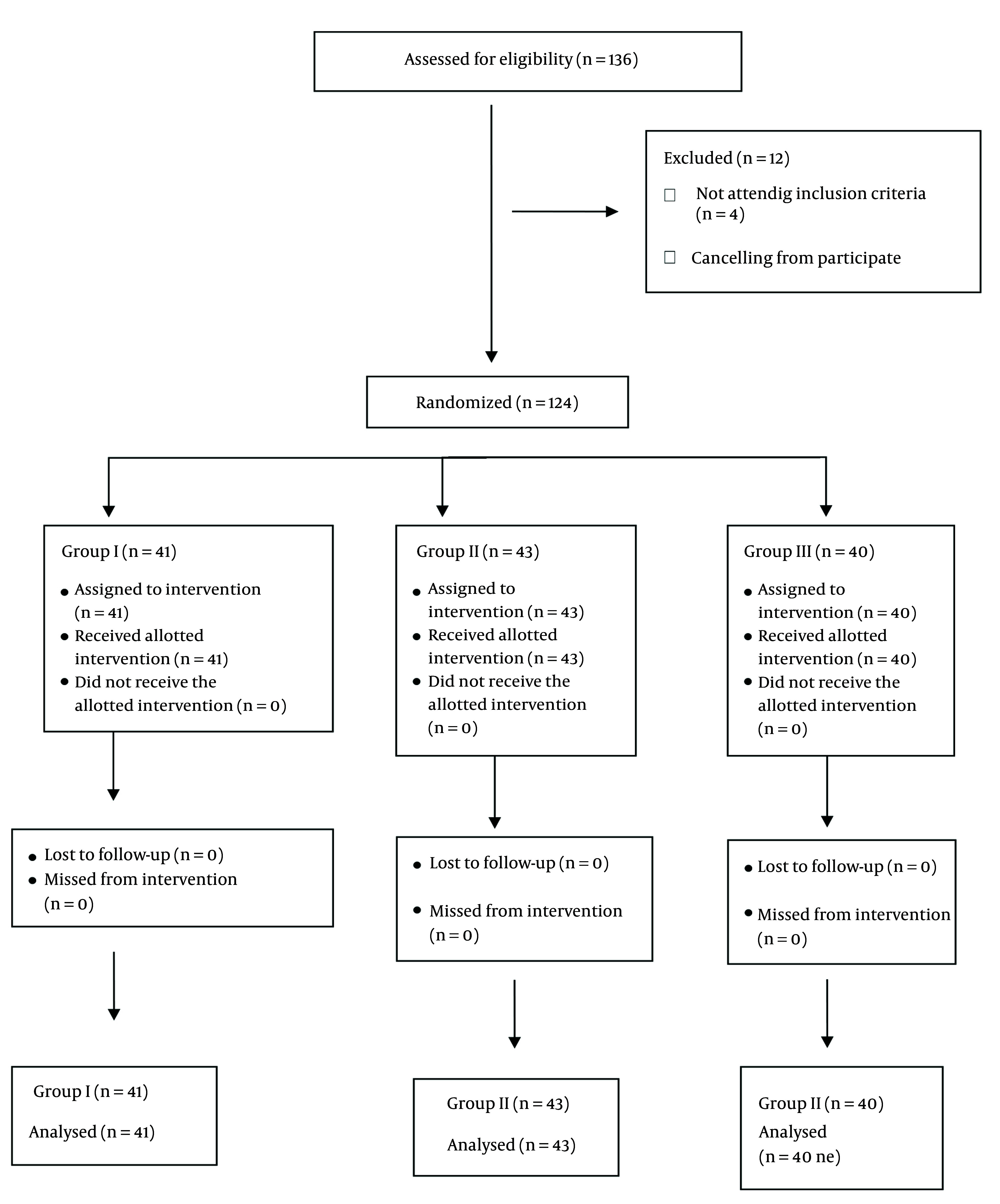
CONSORT flow diagram

The current randomized, double-blind design study was conducted in the intensive care unit of Tadawi Hospital, Dammam, KSA, from December 2022 to May 2024. The hospital ethical committee approved the study under the number 382/64. The study was registered on the ClinicalTrials.gov PRS under the number NCT06687187. The study included 124 patients aged between 20 and 60 years requiring enteral feeding via nasogastric (NG) or orogastric (OG) tubes. Participants were categorized into three groups (Figure 1): Two groups (group I, n = 41) and (group II, n = 43) received varying doses of neostigmine (1 mg and 2 mg respectively). In contrast, a control group (group III, n= 40) received 10 mL of normal saline. Additionally, all participants in the study received an intravenous administration of 10 mg of metoclopramide.

The inclusion criteria encompassed patients with a GRV > 120 mL, a normal heart rate, and the absence of comorbidities such as diabetes and renal failure. Patients exhibiting new-onset arrhythmias or heart block, hypotension (systolic blood pressure less than 60 mmHg), experiencing active gastrointestinal bleeding or receiving prokinetic medications 8 - 12 hours before the intervention, patients with a history of surgery in the gastrointestinal system in the past two weeks or history of extrapyramidal manifestations, patients with electrolyte imbalance, pregnant patients, were excluded from the study. The GRV was measured every 3 hours before enteral feeding. In addition, all patients were monitored regularly to prevent adverse effects.

Both intensivists and patients were kept blind to the drug categorizations. After the signature of the informed consent from the patient or the next of kin, 124 eligible patients were randomly categorized into three groups through sealed envelopes, as shown in Figure 1. In all three groups, 10 mg of metoclopramide was given slowly intravenously over one minute. In groups I, II, and III, 1 mg, 2 mg of neostigmine, and 10 mL of normal saline were injected, respectively.

All participants received enteral feeding with the same formula through boluses every 2 hours in 200 mL via NG or OG tubes. Aspiration precautions were kept for all patients by keeping the head of the bed up to a 30 to 45-degree angle. To check the GRV, aspiration through NG or OG tubes was done before the next due bolus of feeding. Throughout the study, all patients’ enteral feeding data were documented in a pre-steered census and determined using SPSS 25 software.

### 3.1. Sample Size

The possessed data was interpreted using the PASS program, which set the alpha error at 5% and the power at 80%. A recent study by Moshari et al. (12) indicated that the mediocre residual volume immediately following the injection of both neostigmine (1 mg) and metoclopramide was 62 mL, and 3 hours after the injection; it was 42 mL. Based on this, the required sample sizes for the three groups were 41, 43, and 40 for groups I, II, and III, respectively.

## 4. Results

We used immunoassay to determine pepsin in the collected tracheal aspirations. Previously, it was found that the specificity of this test was 100%, and the sensitivity was 93% in animal models (13). We did cryopreservation of the collected sample within one hour of collection for subsequent investigation. This assay can identify pepsin in an amount as low as 1 μg/mL. The same biochemist had read the gels and did not know all the patient’s details and clinical scenarios. All results were determined as either positive or negative results, so positive pepsin results were considered indicators for aspiration of gastric contents.

We conducted this double-blind trial on patients admitted to the intensive care unit at the age of 40 ± 19.5 y (average 20 - 60 years). We did not find any significant difference between all groups regarding demographic parameters and prognostic data, as shown in Table 1. 

**Table 1.** Demographic and Prognostic Data Among All Groups ^a^

Data	Group I	Group II	Group III	P-Value
**Age**	33.5 ± 23.4	39.1 ± 22.5	34.5 ± 20.10	0.892
**Gender**				
Male	22 (53.6)	20 (46.5)	19 (47.5)	0.456
Female	19 (46.3)	23 (53.4)	21 (52.5)	0.513
**BMI**	24.7 ± 5.13	27.3 ± 4.81	25.3 ± 4.23	0.873
**MAP**	76.3 ± 12.5	78.4 ± 13.9	69.33 ± 13.5	0.386
**HR**	88.60 ± 12.20	86.34 ± 15.60	83.48 ± 14.80	0.231
**SOFA score**	4.28 ± 3.4	4.81 ± 3.1	4.67 ± 3.44	0.588
**APACHE score**	16.557 ± 4.56	16.3 ± 4.43	15.4 ± 5.33	0.745

Abbreviation: BMI, Body Mass Index.

^a^ Values are expressed as mean ± SD or No. (%).

As shown in Table 2, there was no significant variation among all groups regarding laboratory markers.

**Table 2.** Laboratory Markers Among All Groups ^a^

Data	Group I	Group II	Group III	P-Value
**Hb**	10.2 ± 2.34	11.1 ± 2.10	10.8 ± 2.81	0.431
**Mg**	1.95 ± 0.42	1.89 ± 0.27	1.87 ± 0.58	0.297
**K**	3.69 ± 0.29	4.1 ± 0.51	3.89 ± 0.51	0.351
**Na**	137.2 ± 4.21	138.4 ± 3.22	138.5 ± 3.22	0.076
**Cl**	97.6 ± 3.33	98.3 ± 4.41	99.3 ± 4.61	0.341

^a^ Values are expressed as mean ± SD.

Regarding the GRV, we can say there was a significant variation among the three groups with a level of significance of 0.0001, as shown in Table 3. 

**Table 3.** Varied Investigations Among Three Groups on the Gastric Residual Volumes

Data	Values	f	Hypothesis df	P-Value
**The remaining volume in the stomach**				< 0.00001
Roy's largest root	2.176	94.632	141	
Hoteling's trace	2.176	94.632	141	
Pillai's trace	0.649	94.632	141	
Wilks' lambda	0.240	94.632	141	
**The remaining volume in the stomach in relation to the group**				< 0.00001
Roy's largest root	0.94	30.558	135	
Hoteling's trace	1.040	11.554	412	
Pillai's trace	570	8.041	417	
Wilks' lambda	0.465	9.831	362.10	

**Figure 2.**
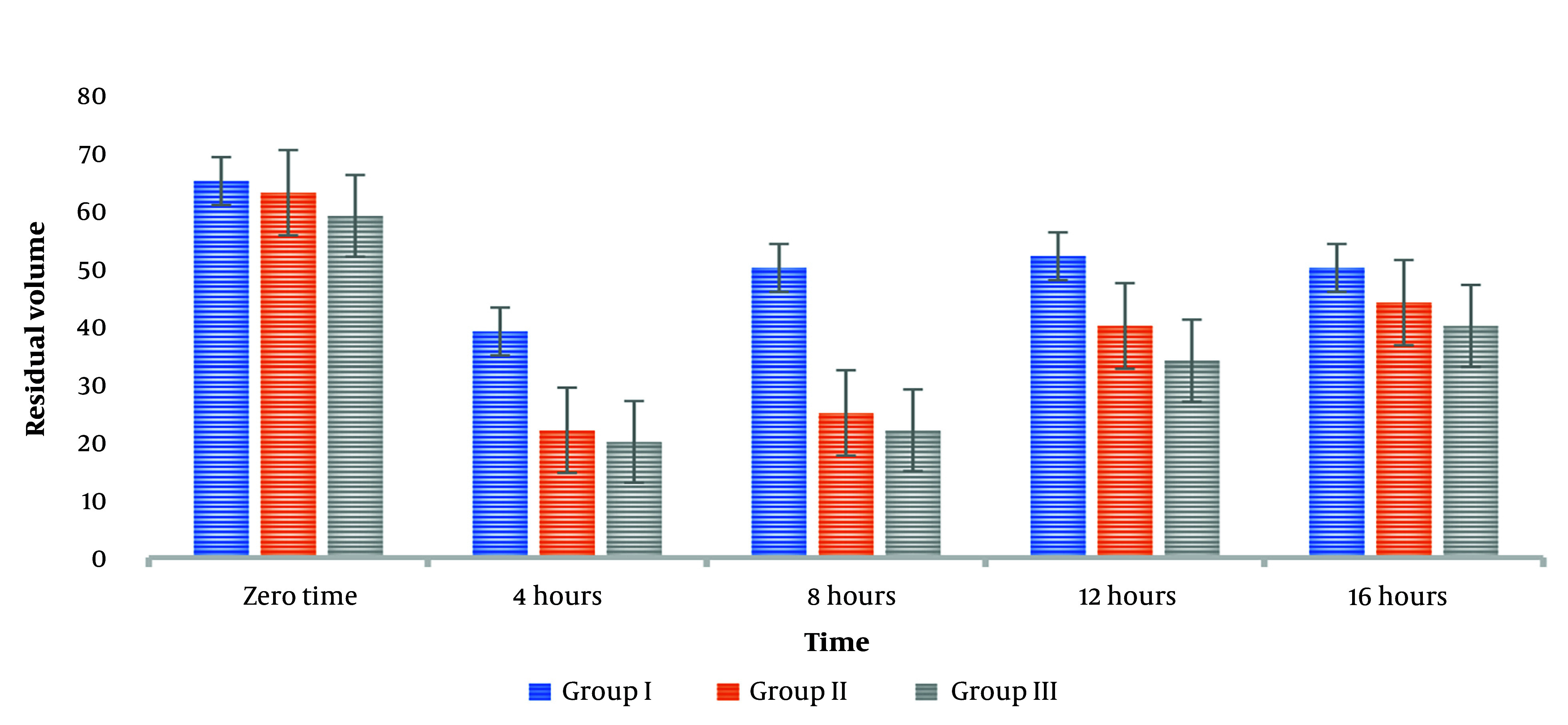
Variations in gastric residual volumes (GRV)

The current study's findings were that metoclopramide, used alone, did not profoundly alter the GRV at various time intervals. However, the administration of neostigmine resulted in a significant reduction in GRV at 3 and 6 hours post-injection. Furthermore, administering 1 mg of neostigmine alongside metoclopramide immediately reduced the mean GRV to 61.451 mL. After three hours, the volume in the cylinder further decreased to 40.325 mL. On the other hand, GRV increased six hours post-injection, indicating that the drug combination resulted in a short-term effect, as shown in Figure 2. 

This study found that doses of neostigmine (1 mg and 2 mg) may be more effective in reducing GRV compared to metoclopramide alone. However, this reduction did not correlate with any alterations in the characteristics or laboratory values of the four groups, suggesting that the severity of the disease did not influence the efficacy of the drugs.

**Figure 3.**
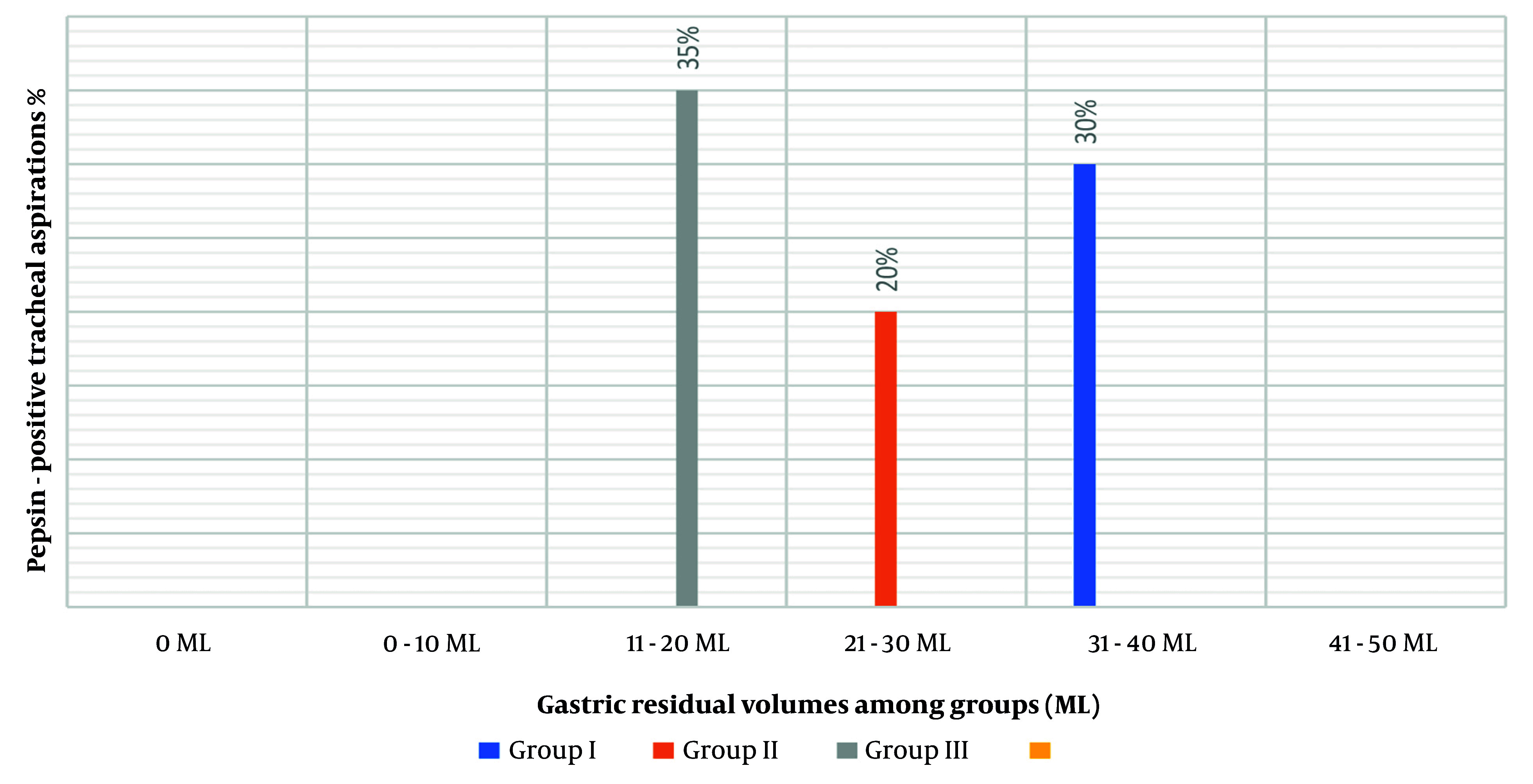
Percent of pepsin-positive tracheal aspirations following gastric residual volume (GRV)

Figure 3 illustrates the proportions of secretions contributing to aspiration affiliated with the continuity of enteral feeding and the amount of GRV ranging from 0 ml to more than 250 mL of aspirates. The percentage of secretions contributing to aspiration when the GRV was between 0 mL and 50 mL was relatively high (30.4%). Nevertheless, the rate of aspiration increased as GRV increased (32 mL in group III consistent with a 35% possibility of pepsin-positive tracheal secretions, 18 mL in group II consistent with a 20% possibility of pepsin-positive tracheal secretions, and 25 mL in group I consistent with a 30% possibility of pepsin-positive tracheal secretions), with a P-value of 0.122.

## 5. Discussion

Our study's idea was to compare the effect of two different doses of neostigmine in conjunction with metoclopramide on the amount of GRV among critically ill patients on enteral feeding either by NG tube or OG tube. Based on the study data, there was no significant variation among the three groups regarding demographic data like age, Body Mass Index (BMI), or vitals like blood pressure or heart rate. We found no significant variation among the three groups regarding laboratory data of APACHE or SOFA scores.

Rahat-Dahmardeh et al. studied the efficacy of both metoclopramide and neostigmine on the GRV of ICU patients on enteral feeding. They concluded that using neostigmine when considering SOFA status and other demographic factors improved the GRV in those patients compared with metoclopramide alone (1). In our study, the correlation of the negligible amounts of GRV among variable hours of the day in each group independently displayed that the quantity of the GRV in all timings has significant differences among groups. This can be explained by the good prokinetic effects of neostigmine and metoclopramide.

 However, the correlation of the average GRV as a comparison of all groups, regardless of the factor of time, demonstrated that there was no significant variation regarding the effects of study medications on the quantity of GRV.

The average findings of GRV at variable timings through the daytime demonstrated that the quantity of GRV at all timings has a significant variation between groups. We can explain this high considerable difference by the gastric volume following injection immediately and after 3 hours, and the minimal considerable difference of the gastric volume following injection immediately and after 12 hours was 3.562. These findings of the average findings of GRV demonstrated that metoclopramide as a solo drug failed to affect the g GRV at variable times because the difference among all groups is approximately the same.

 On the other hand, adding two doses of neostigmine considerably affected the quantity of GRV, especially after 3 and 6 hours following neostigmine injection. This considerable reduction is attributed to the effects of neostigmine doses. However, the fact that the GRV began to increase again after 6 hours is still alarming. This indicates that neostigmine is effective in the short term. Nevertheless, it may necessitate additional doses or other forms of intervention to be sustained over the long term.

 Earlier research in this field has proved that there were no documented significant complications related to neostigmine use, but at the same time, it leads to significant improvements in the patient’s GRV within 12 hours of use (3). At the same time, it should be known that all patients in all dosing groups of neostigmine recovered completely, as described by Gholipour et al.; they compared the use of both neostigmine alone and metoclopramide alone in mechanically ventilated patients on enteral feeding in ICU and concluded that the neostigmine group had a significant lower GRV than the metoclopramide group without considerable side effects (2). In our study, we kept all our patients under close monitoring and observation for any study drug side effects, and we found no incidence of any considerable side effects.

 Our results are based on prior research in this field, demonstrating that neostigmine enhances bowel movement in postoperative patients (6). This reduction in GRV during the 3rd and 6th hours post-administration may mitigate the risks associated with delayed gastric emptying that comprise VAP when utilized to improve feeding tolerance.

 We did not observe any significant link between GRV and incidence of aspiration, as reported in other studies (5). Aspiration happened even with low-volume aspirations. On the other hand, we found that the incidence of aspiration significantly increased as GRV increased, which may be attributed to some degree of gastroesophageal regurge as described by Xin et al (7). They found a considerable relationship between increasing GRV (aspirated blindly) and gastroesophageal reflux in 19 patients in critical care units. However, high GRV may correspond with other predisposing factors like intestinal dysmotility, low conscious level (GCS less than 8), low head of the bed < 30^°^, and impaired gastric emptying; we observed that high GRV possesses an absolute effect on the incidence of aspiration when compared with other risk factors.

### 5.1. Conclusions

According to the results of the double-blind controlled clinical trial, we conclude that the combined administration of neostigmine and metoclopramide can effectively reduce GRV in ICU patients receiving enteral nutrition. The study’s results indicated that metoclopramide alone did not significantly affect the GRV. However, the addition of neostigmine yielded positive outcomes, particularly three and six hours post-injection. The findings of this study offer valuable insights for improving patient feeding tolerance and reducing deterioration in the ICU. Consequently, subsequent research should be conducted to determine the appropriate dosing intervals and the behavioral effects of neostigmine in reducing GRV.

### 5.2. Recommendation

Future studies are needed to find other pharmacological and non-pharmacological modalities to decrease the risk of increased GRV and, hence, aspiration.

## Data Availability

The dataset presented in the study is available on request from the corresponding author during submission or after publication.

## Clinical Trial Registration Code:

NCT06687187.
